# *Anisakis simplex*: The Exclusive Member of Anisakidae Family Infecting Fish Consumed by Humans in Chile Is a Mosaic of Allergens

**DOI:** 10.3390/ijms27135922

**Published:** 2026-06-30

**Authors:** Juan San Francisco, Alejandro Ávalos, Sebastián Brito, Kurt Montoya, Sebastián Zambrano, Nicolás Vivanco, Sebastián Arenas, Carolina Aliaga, Felipe Carter, Gonzalo Pastén, Bessy Gutiérrez, Kyung-Mee Moon, Rafael F. de Almeida, Leonard J. Foster, Jorge González

**Affiliations:** 1 Molecular Parasitology Unit, Medical Technology Departament, Faculty of Health Sciences, University of Antofagasta, Antofagasta 1240000, Chile; juan.sanfrancisco@uantof.cl (J.S.F.); alejandro.avalos@uantof.cl (A.Á.); sebastian.brito.vega@ua.cl (S.B.); kurt.montoya.rojas@ua.cl (K.M.); sebastian.zambrano.medina@ua.cl (S.Z.); nicolas.vivanco.torrejon@ua.cl (N.V.); sebastian.arenas.rojas@ua.cl (S.A.); carolina.aliaga15@gmail.com (C.A.); felipe.carter.garay@ua.cl (F.C.); gonzalo.pasten.quezada@ua.cl (G.P.); bessy.gutierrez@uantof.cl (B.G.); 2 Departament of Biochemistry and Molecular Biology, Michael Smith Laboratories, University of British Columbia, Vancouver, BC V3T1Z4, Canada; kyungmee@mail.ubc.ca (K.-M.M.); foster@msl.ubc.ca (L.J.F.); 3 Laboratório de Biologia Molecular e Sistemica de Tripanossomatídeos (Labtryp), Instituto Carlos Chagas Fiocruz (ICC-Fiocruz), Curitiba 81310-020, Paraná, Brazil; alosteria@gmail.com; 4 Center for Immunology and Biomedical Biotechnology of Antofagasta, University of Antofagasta, Antofagasta 1240000, Chile

**Keywords:** *Anisakis simplex*, fish infections, proteomics, allergens, apolipophorin

## Abstract

This study aims to determine the prevalence of infection by larvae from the *Anisakidae* family in fish commonly consumed in the north of Chile. Then, 2968 specimens belonging to 22 different fish genera were studied. *Anisakis* spp. third-stage larvae were collected and used for PCR and proteomics studies. *Trachurus murphyi* was the most parasitized species (51.6%), whereas *Scomber japonicus* (21.3%) and *Isacia conceptions* (9.5%) were also found parasitized. PCR studies showed that the only species detected was *Anisakis simplex*. By LC-MS/MS, we identified a total of 8119 peptide precursors, which correspond to 1919 proteins. Gene Ontology analysis indicated that, among molecular functions, catalytic and binding activities were the most highly expressed. Among biological processes, cellular and metabolic processes were the most highly expressed, while among cellular components, cellular anatomical entities and complex-containing proteins were the most highly detected. By in silico analyses, novel putative allergens were detected through comparative analyses with related genera. Among them, apolipophorin is proposed as a potential new allergen. These findings are of relevance for advancing the understanding of allergen–host immune system interactions. Proteomics and bioinformatics studies strongly suggest that *A. simplex* is a mosaic of allergens whose implications for public health must be properly evaluated. Finally, a One Health approach is proposed to mitigate *Anisakidae* infections by integrating multisectoral prevention across human and animal interfaces while concurrently preserving aquatic ecosystem integrity.

## 1. Introduction

Anisakids are nematode helminths belonging to the family Anisakidae, which includes the genera *Anisakis*, *Pseudoterranova*, *Contracaecum*, and *Hysterothylacium* [1]. These genera are geographically distributed throughout the world’s oceans across all continents. Consequently, the larvae of the Anisakidae family found in various fish species commonly consumed by humans represent a potential public health risk. However, at present only three *Anisakis* species are of greatest clinical relevance in humans: *Anisakis simplex* sensu stricto, *Anisakis pegreffii*, and *Anisakis physeteris* [2,3].

These helminths have a complex life cycle involving multiple hosts. The adult stages of *Anisakis* spp. are found in the stomach of marine mammals, where they become embedded in the mucosal tissues. The unembryonated eggs produced by adult females are released via the feces of these mammals and subsequently embryonate in the ocean, developing into first-stage larvae (L1) within the eggs. The larvae then molt and successively develop into second- and third-stage larvae (L2 and L3). The L3 larvae hatch from the eggs and swim freely until they are ingested by krill, in which they continue to develop to the L3 stage, the infective form for fish and squid. Through predation, the larvae are transferred to these hosts [4].

Following fish capture and once the host dies, the larvae migrate from the intestine to the abdominal cavity and eventually into muscle tissues, where they grow to lengths of up to 3 cm. Humans become infected by consuming raw or undercooked marine fish and cephalopods containing third-stage larvae. This makes humans accidental hosts, as the worms cannot reproduce or reach the adult stage, and they typically die within approximately three weeks [5].

Anisakidosis, predominantly associated with *A. simplex* and *A. pegreffii*, exhibits a global incidence of 0.32/100,000 [6]. Historically, 90% of 20,000 worldwide cases were localized in Japan, which reports 2000–3000 annual infections [7,8]. In Europe, marked discrepancies exist between confirmed cases and risk projections; for instance, Spanish hospital records cited 2471 cases over 18 years, while models estimate the possible incidence could reach 20,978 annual infections [9]. Seroprevalence in asymptomatic populations fluctuates from 0.4% in Norway to 22.1% in Spain, escalating to 46.4% among occupationally exposed fish industry workers [10,11]. Allergic anisakidosis prevalence peaks in Portugal and Norway at 18.45–22.50% [12]. Ultimately, the disease remains significantly underreported globally due to non-standardized methodologies and non-specific clinical presentations [4]. However, these parasites can cause a gastric disease in humans known as anisakiasis [13]. Anisakiasis (or anisakidosis) is an emerging, cosmopolitan, and highly underdiagnosed disease [14]. Its presence in humans results in several gastrointestinal clinical manifestations, including acute gastric, chronic gastric, and acute intestinal anisakiasis [15]. Invasive intestinal anisakiasis represents a severe manifestation, characterized by luminal stenosis potentially progressing to obstruction. Critical complications include intestinal perforation, peritonitis, and intussusception [16,17,18]. Furthermore, ectopic anisakidosis involves extra-gastrointestinal larval migration to visceral organs, inducing eosinophilic granulomas or tumorous formations that mimic internal malignancies [19,20,21,22]. The diagnosis of gastrointestinal anisakidosis necessitates a comprehensive clinical history focusing on epidemiological data and recent raw fish ingestion. Gastric manifestations typically occur within hours and are definitively diagnosed via upper endoscopy by identifying filiform larvae embedded in the inflamed mucosa [23,24]. Conversely, intestinal involvement presents diagnostic challenges due to delayed symptom onset and frequent anatomical inaccessibility, often mimicking acute surgical pathologies. Radiographic findings, such as CT-detected localized bowel edema or ultrasound demonstrating the characteristic “corn sign,” serve as critical diagnostic adjuncts [23,24]. While invasive, exploratory laparotomy may be utilized for confirmation in cases where clinical presentations are non-specific [23,24]. Ultimately, parasitological examination of extracted larvae using PCR, Western blots or serological detection of specific recombinant allergens provides the definitive genus-level identification [25,26,27,28,29,30].

Additionally, allergic anisakiasis has been described, characterized as an IgE-mediated hypersensitivity reaction [31]. Three forms of allergic anisakiasis are recognized: (i) gastroallergic anisakiasis, involving both gastric symptoms and allergic reaction; (ii) classical allergic reactions, in which patients develop clinical manifestations ranging from mild to severe, such as urticaria, asthma, dermatitis, rhino-conjunctivitis, and, in some cases, anaphylaxis; and (iii) a proposed form occurring in healthy sensitized individuals or asymptomatic patients who exhibit elevated anti-*Anisakis* IgE levels, likely due to a previous subclinical and undiagnosed infection without overt allergic symptoms [32]. To date, *A. simplex* remains the main member of the *Anisakidae* family demonstrated to induce allergic responses in humans [33]. However, more recent evidence has also suggested the allergenic potential of *Pseudoterranova decipiens* [34,35].

Several allergens have been described in the literature, designated as Ani s 1–14. According to various reports, the most clinically relevant allergens include the proteins Ani s 1, Ani s 2, Ani s 3, and Ani s 4 [36]. Then, within hours after consuming raw or improperly cooked fish infected with *Anisakis*, patients may develop a type I hypersensitivity reaction, whose severity ranges from cutaneous involvement with urticaria and angioedema to anaphylactic shock or asthma [15]. Infections caused by *Anisakis* spp. are considered emerging diseases, and according to the European Food Safety Authority (EFSA), the parasite has been classified as a biohazardous organism [7,37].

In Chile, human anisakiasis was first reported by Sapunar et al. (1976) [38]. Since then, human cases have been sporadically documented. Most reported cases correspond to gastric anisakiasis caused by larvae of *P. decipiens* [39,40]. However, only a limited number of studies have been published on this topic, and the epidemiological status of infections caused by larvae of the *Anisakidae* family in fish commonly consumed by humans remains poorly understood. No extensive studies have been conducted to determine which *Anisakis* species are most prevalent in Chile or to characterize their protein expression profiles and allergen repertoires. Likewise, no information is available regarding allergic reactions induced by helminths of the *Anisakidae* family, and the prevalence of *Anisakis* infection in commercially consumed fish is not well known.

Therefore, determining the prevalence of *Anisakis* spp. in fish commonly consumed in the city of Antofagasta is essential for generating reliable knowledge that may guide the design of control and prevention strategies for this parasitosis. In this study, we present a molecular epidemiology, proteomic, and bioinformatic analysis demonstrating that *A. simplex* is the only prevalent species, infecting primarily Chilean jack mackerel (*Trachurus murphyi*) and, to a lesser extent, other fish species such as Chub mackerel (*Scomber japonius*) and Cabinza grunt (*Isacia conceptionis*). *A. simplex* exhibits a complex proteome in which, in addition to the traditional allergens already described, we identified a substantial number of allergenic proteins not previously reported in the literature, including apolipoprotein. Furthermore, we advocate for the implementation of a One Health framework. One Health constitutes an integrated and unifying paradigm aimed at sustainably optimizing the health of humans, animals, and ecosystems. This framework recognizes the fundamental interdependence existing between anthropogenic populations, domestic and wild biota, and their shared environmental matrices. By fostering multisectoral collaboration, the approach mitigates complex biosecurity threats, including zoonotic emergence and food safety vulnerabilities, while preserving ecological integrity [41].

## 2. Results

### 2.1. Parasitism by Larvae of the Anisakidae Family in Commonly Consumed Fish and PCR Determination

In this study, samples were collected from the abdominal cavity and viscera of fish species from the Antofagasta region in northern Chile. The parasites were identified morphologically based on the fundamental macroscopic and microscopic characteristics of third-stage larvae (L3) of the genus *Anisakis*. These features included a cylindrical body measuring approximately 12.30–18 mm in length and 0.34–0.45 mm in width. A total of 987 helminths were collected, all of which corresponded to L3 *Anisakis* larvae, displaying diagnostic structures such as the mucron at the posterior end, the excretory pore, and the ventricle. Nematode larvae were detected in only six of these species (Table 1). Chilean jack mackerel (*Trachurus murphyi*) was the most heavily infected species, with 62 out of 120 examined specimens testing positive for *Anisakis* larvae (51.6%). In contrast, Chub mackerel (*S. japonicus)* showed a much lower parasite burden, with 10 of 47 specimens infected (21.3%), followed by Cabinza grunt (*Isacia conceptionis*) with 4 of 42 (9.5%), Painted comber (*Paralabrax humeralis*) with 3 of 87 (3.5%), and Palm ruff (*Seriolella violacea*) with 2 of 148 infected specimens (1.4%). In bonito (*Sarda chilensis*), larvae were found in only 1 of 167 individuals collected (0.60%) (Table 1).

**Figure 1:**
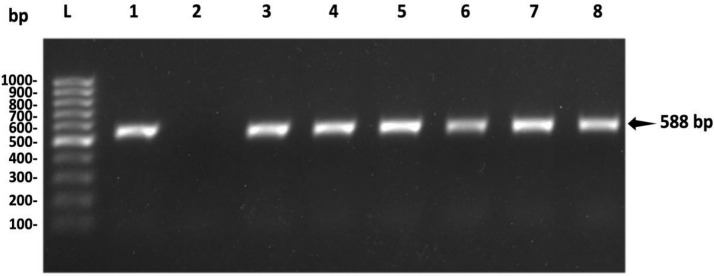
Polymerase chain reaction (PCR) for the identification of *Anisakis simplex*. Larvae collected from fish captured on the coast of Antofagasta. (L), molecular weight marker. Line 1, positive amplification control for *A. simplex*; line 2, negative amplification control for *A. simplex*; lines 3 to 8, amplification of *A. simplex*.

All 987 larvae extracted were submitted to conventional PCR in order to identify the genus and species of the larvae collected. Of the total parasites evaluated by PCR, all of them (100%) were identified as *A. simplex* (Figure 1).

### 2.2. Global Proteome Identification

**Figure 2:**
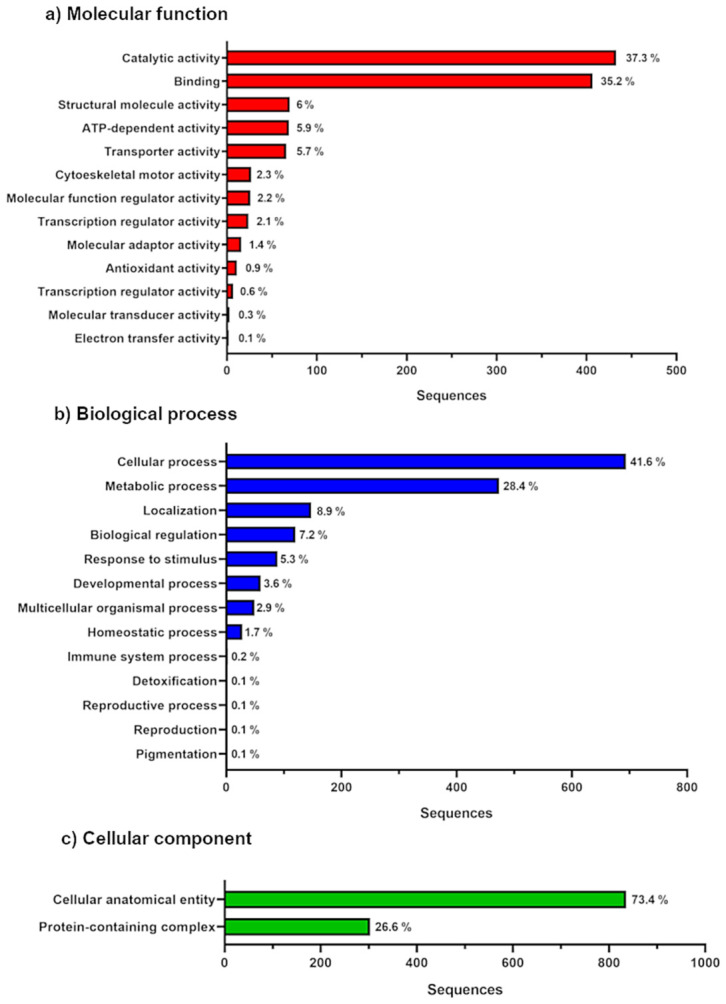
Gene ontology analysis combined with quantitative data for expressed proteins in *A. simplex*. Expression proteins in *Anisakis simplex* associated with (**a**) molecular functions, (**b**) biological processes, and (**c**) cellular components. Only ontologies with *q*-value ≤ 0.05 are presented (Benjamini–Hochberg corrected).

Mass spectrometry analysis identified 8119 peptide precursors from *A. simplex* (Supplementary Table S1), which correspond to 1919 identified proteins (Supplementary Table S2), which corresponds to a total of 1615 gene entries whose GO term annotations were determined using the PANTHER classification system (Figure 2). Among these, the most represented categories within molecular function were catalytic activity (GO:0003824), accounting for 37.3%, and binding (GO:0005488), accounting for 35.2%. Similarly, within biological processes, the most common categories were cellular processes (GO:0009987) with 41.6% and metabolic processes (GO:0008152) with 28.4%. Finally, regarding cellular components, 73.4% of proteins were associated with the category “cellular anatomical entity” (GO:0110165), whereas 26.6% were associated with “protein-containing complex” (GO:0032991) (Figure 2).

Bioinformatic analyses were also conducted using the DAVID platform to determine functional gene classifications and enrichment scores for specific groups. The DAVID functional annotation clustering analysis yielded a total of 140 gene clusters (Supplementary Table S3), with the two clusters displaying the highest enrichment scores being 17.25 and 15.44.

These clusters contained genes with enriched functional annotations corresponding to structural constituents of ribosomes, including ribosomal proteins and ribonucleoproteins in Cluster 1, and translation-related components, ribonucleoprotein complexes, and ribosomal elements in Cluster 2. Subsequently, and with a marked decrease in enrichment score, a cluster with a score of 7.06 was identified, containing genes with functional annotations associated with the parasite’s mitochondrion, including components of the electron transport chain (Table 2).

### 2.3. Allergen Bioinformatic Analysis

From the 1919 proteins identified, a search was conducted to detect putative proteins with allergenic characteristics already described in other species. For this purpose, the AllergenFP tool was used, allowing the identification of a total of 436 proteins with predicted allergenic potential (Supplementary Table S4). Among these, 422 proteins corresponded to allergens previously identified in other species, while 14 proteins were classified as *A. simplex* allergens. Within this latter group, the known allergens Ani s 1, Ani s 2, Ani s 3, Ani s 4, Ani s 5, and Ani s 7 were detected (Table 3). The 422 proteins were subsequently filtered using phylogenetically related organisms to *A. simplex*, resulting in a subset of 105 proteins. These were separated into three groups: (i) potential allergens from human or animal helminths, (ii) allergens from protozoan parasites or allergenic mites, and (iii) a group composed of proteins recognized as potential allergens in organisms such as bees, cockroaches, crustaceans, peanuts, or hazelnuts (Supplementary Table S5).

Of these 105 proteins, duplicate results were removed and a total of 92 proteins were analyzed in AlgPred v. 2.0, to predict and classify allergens, where a hybrid score (ML Score, MERCI Score, BLAST Score) with a threshold value of 1.0 was used to predict highly allergenic proteins in *Anisakis*, and a total of 12 proteins (hybrid score > 1.0) were obtained (Table 4). In addition, further localization analysis performed on the DeepLoc v. 2.1 server showed that 10 proteins have cytoplasmic localization and 2 have extracellular localization (A0A0M3KE55 and A0A0M3IXZ4), all of which are soluble and therefore not membrane proteins (Table 5).

**Figure 3:**
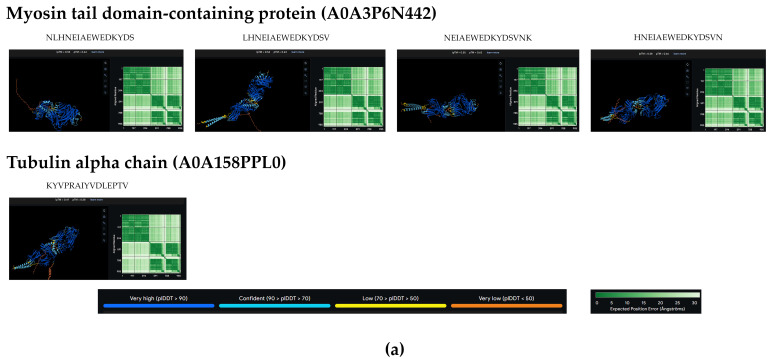

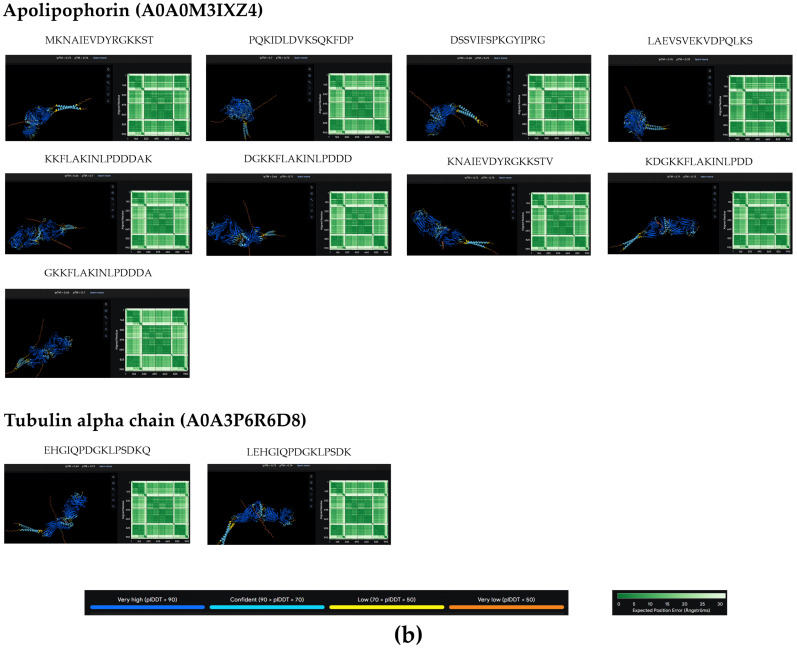

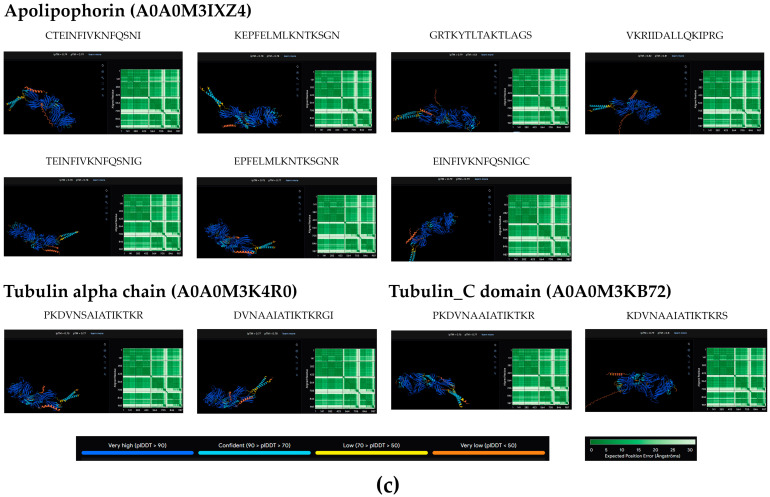
Structural predictions of multiple complexes between different candidate peptides and MHC class II interacting with the T-cell receptor (TCR). Each panel presents a distinct peptide predicted as strong ligands (%rank ≤ 0.02) that were used in structural modeling by AlphaFold in interaction with human MHC II alleles of gene loci DQ (**a**), DR (**b**), and DP (**c**). For each prediction, the three-dimensional structure is colored on the left according to the pLDDT confidence index (blue: high confidence; yellow/orange: low confidence), while the predicted position error (PAE) map is on the right, reflecting the relative reliability between different structural regions. The pTM (predicted template matching score) and ipTM (predicted interface template matching score) values indicate the overall quality of the complex prediction.

Because the type of response it can generate in humans is MHC-II-dependent, the MHC II ligands were mapped for the 12 allergenic proteins using the NetMHCIIpan-4.3 server. Considering the percentile rank ≤ 0.02, peptide size of 15 mers, and relevant human MHC II alleles of the DR, DQ, and DP gene loci (Figure 3), the analysis identified a total of 27 strong MHC II-binding peptides present in the allergens Myosin tail domain-containing protein (A0A3P6N442), Tubulin alpha chain (A0A158PPL0, A0A3P6R6D8), Tubulin_C domain-containing protein (A0A0M3KB72), and Apolipophorins (A0A0M3IXZ4) allergens (Table 6). Of note is the Apolipophorins (A0A0M3IXZ4) protein, with a total of 16 strong-binding peptides.

**Figure 4:**
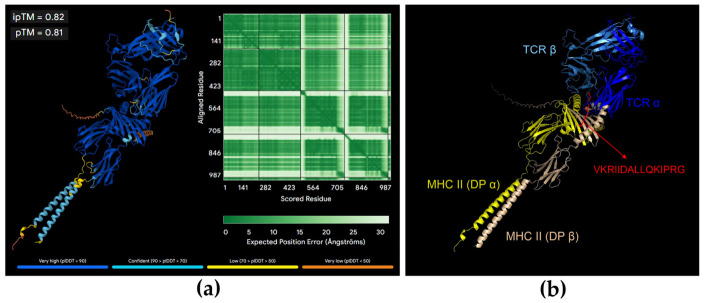
Predicted structural model of the interaction between *Anisakis* allergen peptide and the MHC II–TCR complex. On the left (**a**), structural model with pLDDT confidence coding indicating highly reliable regions (blue) and low-confidence regions (yellow to orange), along with the overall ipTM (0.82) and pTM (0.81) scores. The central predicted position error (PAE) map highlights the relative accuracy between different domains of the structure. On the right (**b**), MHC II–TCR complex is shown, highlighting the TCR α (dark blue) and TCR β (light blue) chains, as well as the α (yellow) and β (beige) chains of MHC II (DP). The peptide VKRIIDALLQKIPRG (in red) is in the MHC-II groove and in direct contact with the TCR, evidencing the molecular recognition interface.

**Figure 5:**
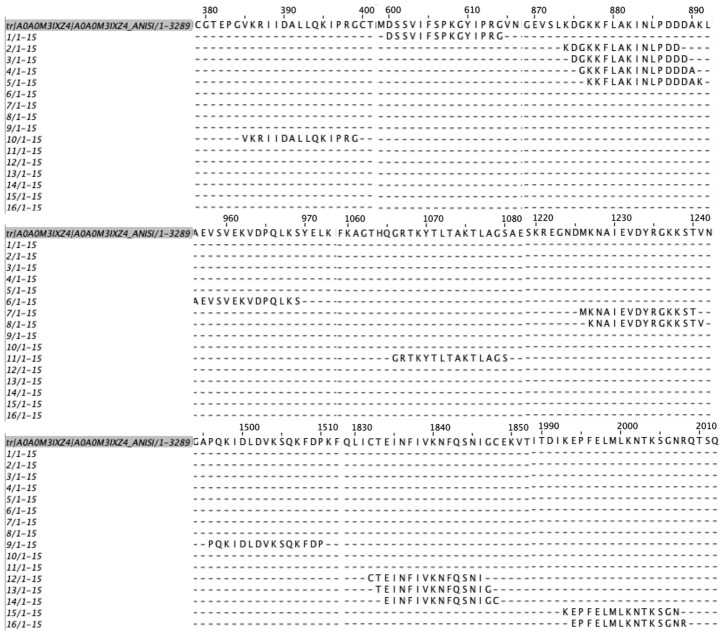
Alignment of identified peptide sequences with the Apolipophorin protein from *A. simplex*. The apolipophorin peptide sequences that bind to MHC-II were aligned to show their location within the protein; these sequences were aligned using Jalview.

The peptides predicted as strong ligands were used in structural modeling with human MHC II BRD (α-β) and TCR (α-β) (Figure 3 and Table 6). The pTM (predicted template matching score) and ipTM (predicted interface template matching score) scores were evaluated, which represent the measurement of structure accuracy, with values for pTM > 0.5 indicating that the complex may be similar to the actual structure and values for ipTM > 0.8 being reliable (ipTM < 0.6 erroneous and in the range 0.6 to 0.8 probably reliable or not) [42,43]. Among the MHC-II-binding peptides, 5 were predicted to bind to the HLA-DQ, 11 to HLA-DR and 11 to HLA-DP isotypes. The best structural model of strong MHC-II ligands, considering the best prediction scores for the individual chain structure (pTM:0.81) and protein interface (ipTM:0.82), was the peptide VKRIIDALLQKIPRG from the Apolipophorins protein with the HLA-DP allele (Figure 4). All peptide sequences corresponding to apolipophorin were aligned with the protein to determine their location (Figure 5).

## 3. Discussion

Members of the Anisakidae family exhibit wide geographic distribution, occurring in fish from all the world’s oceans [15]. However, only certain members of this family possess a high zoonotic potential. Among them, the genus *Anisakis*, particularly the species *Anisakis simplex* and *Anisakis pegreffii*, are the most clinically relevant [15,27,44,45,46,47]. Nevertheless, the scientific literature also reports human infections caused by other species, including *Pseudoterranova cattani* [48,49] and *Pseudoterranova decipiens* [39,50,51].

To date, no studies have been conducted in northern Chile to determine the current situation regarding *Anisakis* spp. infection in commonly consumed fish. In the present study, we report by PCR that 51.6% of Chilean jack mackerel specimens were infected with *A. simplex* larvae. This finding is relevant because it contrasts with previously documented data from central southern Chile. Recently, Muñoz-Caro et al. [49] reported the presence of *Anisakis* in 180 commonly consumed fish belonging to three species collected along the central coast of Chile. Hake (*Merluccius gayi*) and snoek (*Thyrsites atun*) showed 100% infection rates, whereas redbanded seabream (*Brama australis*) exhibited a 35% infection rate [49]. However, in that study, the Anisakidae species identified were *P. cattani* and *A. pegreffii*, while *A. simplex* was not detected [49]. Conversely, another study evaluating 78 “ceviche” portions sold in restaurants in the cities of Valdivia and Niebla revealed that 21.8% of samples contained larvae of *Pseudoterranova* spp. and *Anisakis* spp. [52]. According to the available literature, *A. simplex sensu stricto* has been described mainly in fish captured in the northern hemisphere [15,29,52,53,54]. Meanwhile, a study conducted on fish captured along the central coast of Peru, including jack mackerel, mackerel, hake, and Chilean jack mackerel, showed that *A. pegreffii* was the predominant species, with a prevalence of 53.7% [55]. However, at least one earlier description reports the presence of *A. simplex* in Peruvian and Chilean coastal waters [56]. In that report, identification relied solely on morphological traits, and no molecular methods were employed.

In our study, the second most heavily parasitized fish species was Chub mackerel. This finding is consistent with reports from other authors describing high levels of *Anisakis* spp. infection in this species [57,58,59]. Notably, in our study, only a single *Anisakis* species was detected. This contrasts with findings from other researchers who have reported up to five *Anisakis* species infecting fish within a single geographic region [60]. These findings are epidemiologically relevant, as the high prevalence of *A. simplex sensu stricto* larvae in the musculature of these species represents a public health concern for the Chilean population, particularly considering the widespread consumption of raw or undercooked fish preparations. Likewise, the molecular identification of the infecting species is essential, since among all *Anisakis* species described to date, *A. simplex* has been reported as the primary etiological agent responsible for the majority of human anisakiasis cases [60]. In a previous study conducted on fish captured along the central coast of Chile, anisakid prevalences ranging from 16.7% to 41% were reported in Spanish rockfish (*Sebastes capensis*), Painted comber (*Palabrax humeralis*), and yellowtail amberjack (*Seriola lalandii*) [61]. In the present study, identification was conducted via PCR, owing to its high sensitivity, specificity, and capacity for diagnostic resolution at both the genus and species levels. The application of monoclonal antibodies targeting parasite-specific proteins or allergens may further serve as a complementary diagnostic tool. Conversely, the parasite detection using antibodies present in human sera demonstrates reduced specificity for identification purposes, as the literature documents cross-reactivity between *Anisakis* and other helminths, as well as between certain *Dermatophagoides* and *Anisakis* allergens. Given these constraints, it must also be noted that monoclonal or polyclonal antibodies capable of identifying the parasite or its immunogenic molecules were not available for this study [62,63,64,65].

Various proteomics studies have contributed to deepening our knowledge of the biology of Anisakidae family members [66,67,68]. On the other hand, Palomba et al. (2023) [69] reported the proteomic characterization of extracellular vesicles (EVs) released by third-stage larvae (L3) of *A. pegreffii*, contributing to the understanding of the molecular signals that facilitate interaction between the parasite and its natural and accidental hosts [69]. In our mass spectrometry analysis of *A. simplex* L3, the most represented categories within molecular function were catalytic activity (GO:0003824), accounting for 37.3%, and binding (GO:0005488), accounting for 35.2%. Binding and catalytic functions have also been reported as the primary molecular functions of *A. simplex* L3 by Stryiński et al. (2019) [67]. Among catalytic activities, the most relevant were hydrolase (GO: 0016787), mainly endopeptidases characterized as metalloendopeptidases and cysteine-type endopeptidases. In *A. simplex*, a metalloaminopeptidase was first described by Sakanari and McKerrow in 1990 [70]. Metallopeptidases are involved in the invasion of host tissues by the parasite, as they can degrade the extracellular matrix, and are also involved in the process of ecdysis and digestion of nutrients [71]. On the other hand, high levels of transcripts of a metallopeptidase (nas10) were found in the L3 infecting the muscle of the host fish *Micromesistius potassou* [72]. Several metalloproteases have been shown to be overexpressed in *A. simplex* L3 larvae [73]. Among metalloendopeptidases, we have found neprilysin, which has been associated with intestinal digestion, host invasion, and molting [74]. Metalloprotease and cysteine proteinase activities have also been detected in *A. simplex* and *A. pregreffii* [75,76]. Cysteine proteases in nematodes also appear to play a critical role in parasitic virulence, participating in various processes such as penetration of host tissues, evasion of the immune response, virulence, digestion, embryogenesis, molting, and intracellular digestion [71,77]. Metalloprotease and Cathepsin L activity have been detected in *Anisakis* [75,77].

On the other hand, among the binding expressed proteins, mRNA and actin filament binding were the most expressed. Little is known about the cytoskeleton of *Anisakis*. However, a study using a monoclonal antibody obtained against human intermediate filaments reported cross-reactivity with *A. simplex* [78]. In *A. pegreffii*, actin has been reported to be the main cytoskeletal component present in EVs [69]. A powerful and well-organized cytoskeleton is necessary to keep the parasite moving and penetrate the muscles of infected fish and intermediate hosts. In the same way, the increased synthesis of forms of myosin, actin and troponin has been observed in nematodes as a response to the host immune system and may be related to the attempted reorganization or repair of the cytoskeleton and/or muscle layer in the host, immune-initiated increased mucus production and smooth muscle activity within the intestinal environment [79]. In addition, the anthelmintic drugs currently in use act on the cytoskeleton, so knowledge of these proteins is essential in strategies for developing new drugs or improving those currently in use [80].

In eukaryotes, the mRNA life cycle comprises different stages: transcription, precursor mRNA processing, nuclear export, translation, and degradation. At each stage, mRNAs interact with a specific set of RNA-binding proteins to form mRNA–protein complexes [81,82]. These RNA-binding proteins regulate the activity, localization, and stability of mRNA and influence its transition to the next stage of the life cycle [83]. In *A. simplex*, gene expression patterns have been studied in the L3 and L4 larval stages through various transcriptomic studies [73,84,85], and expression of RNA-binding proteins has been observed in response to exposure to antiparasitic drugs [86,87]. However, the regulation of mRNA expression has not been investigated in depth.

Similarly, within biological processes, the most common categories were cellular processes (GO:0009987) with 41.6% and metabolic processes (GO:0008152) with 28.4%. Among cellular processes, the most highly expressed proteins were chaperone-mediated protein folding (GO:0061077) and chaperone cofactor-dependent protein refolding (GO:0051085). Heat shock proteins (HSPs) are synthesized in response to cellular stress and represent a common mechanism across organisms. They stabilize the conformational structure of newly synthesized polypeptides and facilitate the degradation of those that are misfolded [88]. HSPs have been found to be a common component of nematode EVs and are considered a marker of EVs [89,90]. The presence of HSPs in L3 EVs appears to support their role in mediating and/or buffering the thermal and osmotic stress experienced by the parasite during its life cycle, as it transitions from the marine environment to a natural or accidental homeothermic host. Here, we have reported the higher expression of a 10 kDa mitochondrial heat shock protein and a DNAJ homolog subfamily B member 4. Hsp10 (10 kDa heat shock protein, also known as chaperonin 10 or Cpn10) is a co-chaperone of Hsp60 in the protein folding process. Hsp10 is a homolog of the bacterial protein GroES, which acts as a co-chaperone for Hsp60 (GroEL) in protein folding and assembly processes [91,92]. In eukaryotic cells, Hsp10 homologs are found in organelles such as mitochondria and chloroplasts [93], which originated from bacteria through endosymbiotic processes [94], and play a role analogous to that of their bacterial homologs in the protein folding process [95,96]. On the other hand, members of the HSP40/DNAJ protein family are involved in regulating the molecular chaperone activity of various HSP70 proteins by stimulating their adenosine triphosphate (ATPase) activity [97,98,99].

Therefore, among the metabolic process category, the higher expressed proteins involved were proteolysis, including ubiquitin-dependent protein catabolic process (GO:0006511) and proteasome-mediated ubiquitin-dependent protein catabolic process (GO:0043161). The presence and biological function of the ubiquitin–proteasome pathway were first described in human pathogenic protozoa [100,101,102]. In human parasitic helminths, ubiquitin–proteasome-dependent proteolysis and its biological significance have also been reported [103,104,105]. However, in members of the Anisakidae family, ubiquitin–proteasome pathway components have already been identified in proteomics and transcriptomics studies [72,73,106], but the biological role of this pathway is not well understood.

Finally, regarding cellular components, 73.4% of proteins were associated with the category “cellular anatomical entity” (GO:0110165), whereas 26.6% were associated with “protein-containing complex” (GO:0032991) [69] (Figure 2). Cellular anatomical entity refers to the expression of both cytoplasmic and intracellular organelle proteins. The main cytosolic proteins were the large and small ribosomal subunits, while mitochondrial respiratory complexes were the most highly expressed proteins among “protein-containing complex”. Mitochondrial enzyme genes are highly expressed in *Anisakis* L3 and L4 larvae. It has been reported that L3 of *A. simplex* transcribes genes related to invasion, migration, molting, and survival [36]. Studies in *A. pegreffii* have shown that mitochondrial enzymes are the most highly expressed in L3 larvae [107]. Proteomics studies with *A. simplex* L3 and L4 exposed to glucose concentrations showed that 24 proteins associated with translation and ribosome formation were affected [108]. On the other hand, the large and small ribosomal subunits have previously been reported by Marzano et al. [109].

Using advanced bioinformatics techniques, we initially screened 422 proteins corresponding to allergens in other species along with 14 well-known *A. simplex* allergens, including Ani s 1, Ani s 2, Ani s 3, Ani s 4, Ani s 5, and Ani s 7 (Table 3). These proteins were filtered and categorized into three groups based on their phylogenetic relationship to known allergens in helminths, protozoa, and other organisms such as crustaceans or peanuts (Supplementary Table S5). Following the removal of duplicates, 92 proteins were analyzed using AlgPred v. 2.0. By applying a hybrid score threshold of 1.0, the study identified 12 proteins predicted to be highly allergenic. Localization analysis using DeepLoc v. 2.1 further determined that ten of these proteins were cytoplasmic and two were extracellular; however, all of them were categorized as soluble proteins (Table 5).

Our study was focused particularly on the interaction with the human immune system through MHC II ligand mapping. Among the 12 allergenic proteins, 27 strong MHC II-binding peptides were identified, primarily within the myosin tail domain, tubulin, and apolipophorins (Figure 3). The apolipophorin protein (A0A0M3IXZ4) appears to be of higher interest because it contains 16 of the 27 identified strong binding peptides. When AlphaFold was utilized to generate three-dimensional models of these strong ligands interacting with human MHC II and T-cell receptors (TCR), we observed that the most reliable model was found for the peptide VKRIIDALLQKIPRG from the apolipophorin protein (Figure 4). These findings allow for a more precise prediction of which parasite components could trigger the most severe allergic reactions in humans.

To date, around 14 allergens have been described, and most of them have been detected in parasitic somatic and excreted/secreted products, being recognized by serum antibodies in most individuals affected by allergic anisakiasis. Some of them, such as Ani s 1, Ani s 4, Ani s 5, Ani s 8, Ani s 9, Ani s 10, and Ani s 11, have shown heat stability [110,111,112], which may cause problems even when infected fish is eaten cooked. On the other hand, twenty-eight different allergenic proteins have been classified, and most of them were described for the first time as potential new allergens in *Anisakis* [106]. These researchers showed differences in the allergenic abilities of the *A. simplex* complex species and, using 2D gel electrophoresis, found that *A. simplex* s.s. showed a higher number of immunoreactive protein spots (34 spots) than *A. pegreffii* (11 spots) [106]. In the same way, an approach was used by Fæste et al. [113] in a study based on gel banding patterns and IgE-immunostaining using sera from *A. simplex*-sensitized patients and proteome data obtained by mass spectrometry. Results showed that *A. simplex* proteins were homologous to allergens already characterized in other nematodes, insects, and shellfish [114]. This proteomic investigation of *A. simplex* L3 identified 17 novel putative allergens, including structural proteins such as myosin-4 and enzymes such as enolase and endochitinase [113].

Allergic symptoms, including anaphylaxis, have been reported in approximately 11% of anisakiasis cases in Spain [115]. In Japan, allergy induced by *Anisakis* is a common trigger of acute allergic reactions in adults and represents the second leading cause of anaphylactic shock, accounting for 23% of reported cases [116]. Allergic manifestations have also been described following inhalation or direct contact during the handling of infected fish, leading to rhinoconjunctivitis, dermatitis, and asthma [2,117]. All these clinical responses are triggered by an IgE-mediated immune mechanism.

To date, fourteen *A. simplex* allergens have been identified, designated Ani s 1 through Ani s 14 [118], but the interaction between these allergens and human MHC class II allele key molecules responsible for antigen presentation has been only minimally explored. Previous studies have identified binding motifs from Ani s 2 and Ani s 3 (paramyosin and tropomyosin) associated with alleles such as DRB11502, DQB10601, and DRB1*0404 [119]. Moreover, in a cohort of fish-processing workers in Croatia, PCR-based HLA typing and serological analyses revealed associations involving HLA-DRB1, HLA-DQA1, and HLA-DQB1 [120]. Similarly, little is known about the identification of peptides that participate as T-cell epitopes, as they have only been studied in a few allergens such as Ani s 1, Ani s 5, Ani s 7, and Ani s 13 [121,122,123,124].

Our work suggests that there are proteins not yet identified as *A. simplex* allergens that may generate an MHC class II-mediated allergenic response (Table 5). In silico analysis showed that 27 peptide sequences could be considered potential new allergens because they may react with MHC II alleles, the most notable being the peptide VKRIIDALLQKIPRG (Figure 4), which belongs to apolipophorin and contains a total of 16 probable binding epitopes.

Apolipophorin is a protein consisting of 3289 amino acids found in the *Anisakis* genome. Figure 5 shows the location of the MHC-II-binding peptides within the protein, which helps us understand the position of relevant peptides in its primary structure, such as VKRIIDALLQKIPRG, which is located between amino acid residues 385–400 of the protein. It was initially described as an allergen in *Dermatophagoides farinae*, characterized as an apolipoprotein possessing Mag 1 and Mag 3 sequences that bind to IgE, showing homology with a high molecular weight allergen M-177. This protein is similar to lipid transport apolipoproteins found in insects [125] and was subsequently classified as belonging to group 14 of the main mite allergens [126]. Similarly, apolipophorins have been reported in other insects, such as *Culex quinquefasciatus*, where they represent the main component of lipophorins that mediate the transport of various lipids in the hemolymph and form lipoprotein particles that bind to other lipoproteins and lipids [127].

Research using mass spectrometry has demonstrated allergenic characteristics of apolipophorins in species such as *Acheta domesticus* and *Hermetia illucens* [128]. They have also been identified in other insects such as *Bombyx mori* and *Tenebrio molitor* in studies focused on allergen detection for food safety using proteomic and bioinformatic analysis [129]. In addition, apolipophorin has been identified as a component of venom in the brown widow spider *Latrodectus geometricus* [130]. Although the LLTP superfamily has been described in various species for its allergenic potential to bind IgE, in nematodes this has mainly been associated with vitellogenin, not apolipophorin. Therefore, the allergenic potential of apolipophorin in *Anisakis* is demonstrated here for the first time.

**Figure 6:**
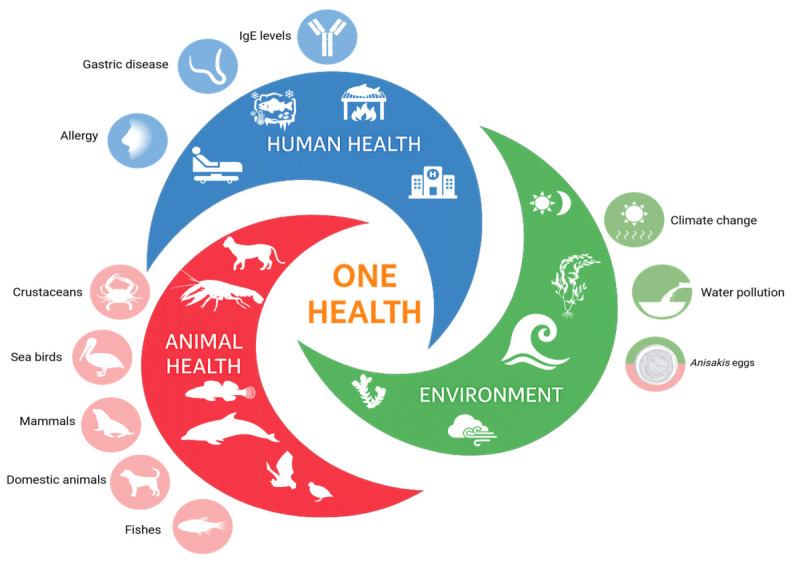
A one health vision for *Anisakis* spp. and anisakiasis.

In this study, we show that a significant percentage of Chilean jack mackerel, a species widely consumed by humans, is parasitized by *A. simplex*. Our proteomics study showed that *A. simplex* L3 larvae possess several previously described allergens as well as potential new allergens suggested by our in silico analysis. This indicates potential health risks for both humans and animals and supports the need for a One Health approach [4,131]. At the human level, measures such as freezing fish or cooking it at temperatures above 70 °C are effective in preventing anisakiasis in its digestive forms. However, the presence of heat-stable allergens means that these measures do not prevent allergic reactions. Therefore, identifying and removing allergens from fish meat is highly necessary. Proteomics has proven effective in detecting proteins and allergens in fish meat [132], but methods to remove them are still lacking. Activated charcoal has been shown to absorb allergens [133,134,135], and modified charcoal nanoparticles that do not alter fish properties could be a potential solution. At the animal level, prevention is limited due to the parasite’s life cycle and host range. Infection in pets such as dogs and cats can be prevented by avoiding raw fish consumption. A major concern is environmental contamination: in Chile, fish waste is often discarded into the sea, feeding seabirds, fish, and sea lions, thus perpetuating the parasite’s life cycle. In the same way, a One Health approach aimed at preventing human and animal infection by Anisakidae while preserving the environment is illustrated in Figure 6.

## 4. Materials and Methods

### 4.1. Collection of Fish Specimens in the Antofagasta Region, Chile, and Extraction of Third-Stage Larvae (L3)

A total of 2968 specimens belonging to the most commercially important fish species sold in the seafood market of Antofagasta, Chile, and captured along the regional coastline were examined. Fish species marketed in the city but originating from other regions of the country were not included in this study. Each fish specimen was dissected to search for anisakid larvae in the abdominal cavity and viscera. Third-stage larvae (L3) were retrieved by carefully removing the visceral organs from the abdominal cavity. All recovered larvae were washed in saline solution (0.85% NaCl) and subsequently prepared for morphological identification under light microscopy.

For every specimen, records were kept including species, body length, and anisakid burden. All anisakid larvae were individually labeled, cataloged, and maintained at cold temperature for transport to the laboratory. For molecular and proteomic analyses, larvae were stored at −80 °C until processing.

### 4.2. Morphological Identification of Larvae of the Family Anisakidae

Morphological identification was based on the following characteristics: (a) position of the excretory pore, (b) tail shape, (c) length and shape of the ventricle, (d) presence, length, and position of the intestinal cecum, and (e) presence or absence of the ventricular appendix. Nematodes were cleared in graded glycerin solutions. Internal structures were examined using a light microscope equipped with a camera lucida. Nematodes belonging to the family *Anisakidae* were identified to the genus level [107,136].

### 4.3. DNA Extraction

Genomic DNA was isolated using the Wizard Genomic DNA Purification Kit (Promega, Madison, WI, USA) from whole individual larvae. Samples were homogenized using a tissue homogenizer (PT 1200 E, POLYTRON^®^, Kinematica AG, Malters, Switzerland) in 600 µL of lysis buffer (0.1 M Tris-HCl pH 7.6, 50 mM DTT, 2% SDS). The homogenate was incubated at 100 °C for 5 min, followed by the addition of 3 µL of RNase and incubation at 37 °C for 30 min. Subsequently, 200 µL of protein precipitation solution was added, the mixture was vortexed for 20 s, and incubated on ice for 5 min [133].

Samples were centrifuged at 16,000× *g* for 4 min, and the supernatant (600 µL) was transferred to a 1.5 mL microtube containing 600 µL of isopropanol. After centrifugation at 16,000× *g* for 1 min, the supernatant was discarded, and the pellet was washed with 600 µL of 70% ethanol, gently inverted several times, and centrifuged again at 16,000× *g* for 1 min. The pellet was air-dried for 10–15 min, resuspended in 50 µL of rehydration solution (10 mM Tris-HCl, 1 mM EDTA), and incubated at 65 °C for 1 h with occasional mixing. DNA quality was evaluated by absorbance at 260/280 nm using a Tecan Infinite M200 Pro spectrophotometer (Tecan Group Ltd., Männedorf, Switzerland) and by agarose gel electrophoresis. Purified DNA was stored at −80 °C until conventional PCR analysis.

#### Conventional PCR Identification of Anisakidae Larvae

PCR assays were conducted as previously described [137] using a final reaction volume of 20 µL on a T100 Thermal Cycler (Bio-Rad, Hercules, CA, USA). PCR products were visualized by agarose gel electrophoresis using SYBR™ Safe DNA Gel Stain (Invitrogen™, Thermo Fisher Scientific, Waltham, MA, USA). Species-specific primers targeting six anisakid species were used (Table 7). In all reactions, a universal type B reverse primer was included (5′-GCCGGATCCGAATCCTGGTTAGTTTCTTTTTCT-3′). Primers were synthesized by Integrated DNA Technologies (Coralville, IA, USA).

PCR products were resolved on 1% (*w*/*v*) agarose gels (UltraPure™ Agarose, Invitrogen, Thermo Fisher Scientific) and stained with SYBR™ Safe (Thermo Fisher Scientific). Genomic DNA from L3 larvae of *Anisakis simplex*, *Anisakis pegreffii*, *Anisakis physeteris*, and *Pseudoterranova decipiens* (provided by Dr. Rubén Mercado, Parasitology Unit, Faculty of Medicine, University of Chile) was used as positive control.

### 4.4. In Gel Digestion

Frozen larvae were homogenized using a Polytron PT 1200 E tissue homogenizer (Kinematica AG, Malters, Switzerland) in lysis buffer (0.1 M Tris–HCl pH 7.6, 50 mM DTT, 2% SDS). Lysates were boiled at 100 °C for 5 min, cooled to room temperature, and clarified by centrifugation at 16,000× *g* for 10 min [138]. Total protein concentration was determined using the Pierce™ 660 nm Protein Assay (Thermo Fisher Scientific).

Protein extracts were processed following an in-gel digestion workflow and digested with trypsin to generate peptides suitable for proteomic analysis [139]. Peptide quantification was performed using the Pierce™ Quantitative Peptide Assay (Thermo Fisher Scientific), and absorbance at 480 nm was measured in a Tecan Infinite 200 PRO microplate reader according to the manufacturer’s instructions.

### 4.5. Reverse Phase-Liquid Chromatography-Tandem Mass Spectrometry (RP-LC-MS/MS) Analysis

Peptide concentrations were measured on a NanoDrop One (Thermo Fisher Scientific) to load approximately 50 ng of peptides on a timsTOF Pro2 (Bruker Daltonics, Billerica, MA, USA) with a CaptiveSpray source coupled to nanoElute UHPLC (Bruker Daltonics) using a PepSep Ten column (10 cm × 75 µm inner diameter, 1.9 µm particle size with 20 µm emitter) heated to 50 °C. Buffer A consisted of 0.1% formic acid and 0.5% acetonitrile in water, and buffer B consisted of 0.1% formic acid and 99.4% acetonitrile in water. A standard 30 min gradient was used, increasing buffer B from 2% to 12% over 15 min and then to 33% over 15 min. The column was washed with 95% B over 8 min. The nanoElute thermostat temperature was set at 7 °C. The analysis was performed at a flow rate of 0.4 µL/min.

The timsTOF Pro2 was set to DIA-PASEF mode with positive polarity for the MS scan window from 100 to 1700 *m*/*z*. The capillary voltage was set to 1800 V, drying gas to 3 L/min, and drying temperature to 180 °C. The MS1 scan was followed by PASEF ramps containing 22 non-overlapping 35 *m*/*z* isolation windows covering the mass range 319.5–1089.5 Da. Ion mobility range (1/K0) was set to 0.70–1.35 V·s/cm^2^, with 100 ms ramp time, 100 ms accumulation time, 100% duty cycle, and 9.42 Hz ramp rate. Collision energy was ramped linearly as a function of mobility from 27 eV at 1/K0 = 0.70 V·s/cm^2^ to 55 eV at 1/K0 = 1.35 V·s/cm^2^.

The acquired data were searched with DIA-NN (PMID31768060) version 1.8.1 with a library-free search method, using UniProt’s *Anisakis simplex* protein sequences (UP000267096) and manually curated common contaminants (226 entries). Other search parameters include Trypsin/P digestion mode with 1 missed cleavage, 1 maximum number of variable modifications, N-terminal M excision, carbamidomethylation of C, and oxidation of M options enabled; peptide length ranged 7–30, precursor charge ranged 1–4, precursor *m*/*z* ranged 300–1800, and fragment ion *m*/*z* ranged 200–1800. Precursor FDR was set to 1%, with 0 Mass accuracy and MS1 accuracy (for the “auto” option of mass tolerance), enabling heuristic protein inference, use of isotopologues, match between run (MBR), and no shared spectra. Protein inference is set as “Protein name from FASTA”, Double-pass mode for neural network classifier, Robust LC (high precision) for Quantification Strategy, RT dependent mode for Cross-run normalization, and Smart profiling mode for Library generation.

The mass spectrometry proteomics data have been deposited to the ProteomeXchange Consortium via the PRIDE (PubMed ID: 34723319) partner repository with the dataset Identifier PXD044267.

### 4.6. Functional Analysis

To assess the biological and molecular functions of the identified proteins, the final list of non-redundant proteins obtained after global identification was classified into the three main Gene Ontology (GO) categories: molecular function, biological process, and cellular component. The Kyoto Encyclopedia of Genes and Genomes (KEGG) database was used to assign the identified proteins to specific biological pathways and cellular mechanisms, applying an adjusted *p*-value < 0.05 as the significance threshold. GO and KEGG enrichment analyses were performed using the PANTHER classification system (Protein ANalysis THrough Evolutionary Relationships, https://pantherdb.org/, accessed on 23 June 2025) [140] and DAVID (https://davidbioinformatics.nih.gov/, accessed on 26 June 2025) [141,142]. GO categorization applied the PANTHER overrepresentation test with Bonferroni correction to adjust the smallest *p*-values associated with enriched GO terms.

### 4.7. Bioinformatic Allergen Analysis

To identify putative novel allergens, the AllergenFP v1.1 platform (https://ddg-pharmfac.net/AllergenFP, accessed on 10 February 2024) [143] was used as an initial screening approach. Subsequently, allergen prediction and classification based on the obtained protein sequences were performed using AlgPred 2.0 (https://webs.iiitd.edu.in/raghava/algpred2/batch.html, accessed on 14 August 2025) [144]. In addition, AlphaFold 3 (https://deepmind.google/technologies/alphafold/, accessed on 15 August 2025) [145] was employed for structural modeling, incorporating MHC class II molecules and human T-cell receptors (TCR α and β). Structural references were based on the Protein Data Bank entry 6TRO [146]. Subcellular localization and membrane-association prediction were conducted using DeepLoc v2.1 (https://services.healthtech.dtu.dk/services/DeepLoc-2.1/, accessed on 2 September 2025) [147]. Finally, to obtain a deeper computational assessment of peptide-binding affinity to MHC class II alleles, the NetMHCIIpan 4.3 tool (https://services.healthtech.dtu.dk/ser-vices/NetMHCIIpan-4.3/, accessed on 11 November 2025) [148] was used.

## 5. Conclusions

This study provides robust molecular and proteomic evidence that significantly advances our understanding of *A. simplex* epidemiology and pathogenicity in northern Chile. The high prevalence of *A. simplex sensu stricto* detected in widely consumed fish species, particularly Chilean jack mackerel, represents a notable geographic and epidemiological shift compared to previous reports from central southern Chile and the southeastern Pacific. This finding underscores the need for updated surveillance strategies integrating molecular diagnostics to accurately identify zoonotic species with high clinical relevance.

From a mechanistic perspective, the proteomic characterization of L3 larvae reveals a complex biological system dominated by catalytic and binding activities, including metalloproteases and cytoskeletal-associated proteins, which are crucial for host invasion, immune evasion, and parasite survival. Furthermore, the identification of stress-response proteins and components of the ubiquitin–proteasome pathway highlights adaptive mechanisms that facilitate environmental transitions and host parasite interactions.

Importantly, the integration of bioinformatics and immunoinformatics approaches enabled the prediction of novel allergenic proteins and MHC-II binding epitopes, with apolipophorin emerging as a key candidate with previously unrecognized allergenic potential. These findings have profound implications for public health, as they expand the repertoire of clinically relevant allergens beyond currently characterized molecules.

Overall, this work emphasizes the necessity of a One Health framework, combining epidemiological monitoring, molecular research, and food safety interventions to mitigate both infectious and allergic risks associated with anisakiasis.

## Figures and Tables

**Table 1:** Infestation by *Anisakis simplex* larvae in fish commonly consumed in northern Chile.

Common Name	Scientific Name	No. of Fish	Infected	Percentage
Chilean jack mackerel	*Trachurus murphyi*	120	62	51.6%
Chub mackerel	*Scomber japonicus*	47	10	21.3%
Cabinza grunt	*Isacia conceptionis*	42	4	9.5%
Painted comber	*Paralabrax humeralis*	87	3	3.5%
Palm ruff	*Seriolella violacea*	148	2	1.4%
Bonito	*Sarda chiliensis chiliensis*	167	1	0.6%
Chilean anchovy	*Engraulis ringens*	1926	0	0%
Chilean morwong	*Cheilodactylus variegatus*	83	0	0%
Yellowtail amberjack	*Seriola lalandi*	74	0	0%
Silverside	*Basilichthys australis*	56	0	0%
Chilean blenny	*Labrisomus philippii*	53	0	0%
Cape rockfish	*Sebastes capensis*	28	0	0%
Chilean sanperch	*Pinguipes chilensis*	25	0	0%
Chilean grunt	*Anisotremus scapularis*	24	0	0%
Bigeye hapuku	*Hemilutjanus macrophthalmos*	16	0	0%
Yellow corvina	*Cilus gilberti*	16	0	0%
Golden kingklip	*Genypterus blacodes*	14	0	0%
Pacific sardine	*Sardinops sagax*	14	0	0%
Atlantic menhaden	*Brevoortia maculata*	11	0	0%
Chilean flounder	*Paralichthys adspersus*	10	0	0%
Snoek	*Thyrsites atun*	4	0	0%
Black kingklip	*Genypterus maculatus*	2	0	0%
Painted sea bass	*Acanthistius pictus*	1	0	0%

**Table 2:** Analyses for DAVID. Bioinformatic analyses using the Database for Annotation, Visualization and Integrated Discovery platform to determine functional gene classifications and enrichment scores for specific groups of *Anisakis simplex* proteins.

**Annotation cluster 1**	**Enrichment Score: 17.25**	**Count**	***p-*** **Value**	**Benjamini**
GOTERM_MF_DIRECT	Structural constituent of ribosome	72	6.4 × 10^−27^	4.1 × 10^−24^
GOTERM_BP_DIRECT	Translation	57	6.9 × 10^−23^	3.9 × 10^−20^
UP_KW_MOLECULAR_FUNCTION	Ribosomal protein	43	8.3 × 10^−12^	5.3 × 10^−10^
UP_KW_MOLECULAR_FUNCTION	Ribonucleoprotein	46	2.7 × 10^−10^	8.8 × 10^−9^
**Annotation cluster 2**	**Enrichment Score: 15.44**	**Count**	***p*** **-Value**	**Benjamini**
GOTERM_BP_DIRECT	Translation	57	6.9 × 10^−24^	3.9 × 10^−20^
GOTERM_CC_DIRECT	Ribonucleoprotein complex	32	4.0 × 10^−13^	3.5 × 10^−11^
GOTERM_CC_DIRECT	Ribosome	29	1.7 × 10^−12^	1-1 × 10^−10^
**Annotation cluster 3**	**Enrichment Score: 7.06**	**Count**	***p*** **-Value**	**Benjamini**
UP_KW_CELLULAR_COMPONENT	Mitochondrion inner membrane	25	8.0 × 10^−11^	9.4 × 10^−10^
UP_KW_BIOLOGICAL_PROCESS	Respiratory chain	14	3.2 × 10^−7^	2.0 × 10^−5^
UP_KW_BIOLOGICAL_PROCESS	Electron transport	13	2.5 × 10^−5^	2.7 × 10^−4^

**Table 3:** *Anisakis simplex* allergens detected by proteomic studies. Of the 1919 proteins identified by LC-MS/MS, 14 *A. simplex* allergens were detected using the AllergenFP tool. These are Ani s1, Ani s2, Ani s3, Ani s4, Ani s5, and Ani s7.

No	ID Protein	Name Protein	ID Similar Allergen	Name Protein	Allergen	Organism
**1**	A0A0M3JUR6	Kunitz/Bovine pancreatic trypsin inhibitor domain protein	UniProtKB: Q7Z1K3	Major allergen Ani s 1	Ani s 1	*Anisakis simplex*
**2**	A0A0M3JKD1	IF rod domain-containing protein	GenBank: AAF72796.1	Paramyosin	Ani s 2	*Anisakis simplex*
**3**	A0A0M3K9P2	Chloride intracellular channel exc-4 (inferred by orthology to a *C. elegans* protein)	GenBank: AAF72796.1	Paramyosin	Ani s 2	*Anisakis simplex*
**4**	A0A3P6NTN9	Myosin tail domain-containing protein	GenBank: AAF72796.1	Paramyosin	Ani s 2	*Anisakis simplex*
**5**	A0A158PP35	Paramyosin (inferred by orthology to a *C. elegans* protein)	GenBank: AAF72796.1	Paramyosin	Ani s 2	*Anisakis simplex*
**6**	A0A3P6S783	Myosin tail domain-containing protein	GenBank: AAF72796.1	Paramyosin	Ani s 2	*Anisakis simplex*
**7**	A0A3P6PH85	Myosin tail domain-containing protein	GenBank: AAF72796.1	Paramyosin	Ani s 2	*Anisakis simplex*
**8**	A0A3P6PGP6	Myosin tail domain-containing protein	GenBank: AAF72796.1	Paramyosin	Ani s 2	*Anisakis simplex*
**9**	A0A0M3J8T0	HOOK domain-containing protein	GenBank: AAF72796.1	Paramyosin	Ani s 2	*Anisakis simplex*
**10**	A0A0M3KCE6	Tropomyosin (inferred by orthology to a *C. elegans* protein)	UniProtKB/Swiss-Prot: Q9NAS5.1	Tropomyosin allergen Ani s 3	Ani s 3	*Anisakis simplex*
**11**	A0A0M3JDX4	Cystatin	GenBank: CAK50389.1	Ani s 4 allergen	Ani s 4	*Anisakis simplex*
**12**	A0A3P6RUB2	SXP/RAL-2 family protein Ani s 5-like cation-binding domain-containing protein	GenBank: BAF75707.1	SXP/RAL-2 family protein 2 isoform 5	Ani s 5	*Anisakis simplex*
**13**	A0A0M3JSH7	Proteasome subunit alpha type-3	UniProtKB: A1IKL2	SXP/RAL-2 family protein Ani s 5	Ani s 5	*Anisakis simplex*
**14**	A0A0M3JZS2	Transmembrane protein	GenBank: ABL77410.1	UA3-recognized allergen, partial	Ani s 7	*Anisakis simplex*

**Table 4:** Allergens identified in other related organisms. The LC-MS/MS analysis identified proteins that are classified as allergens in other species. Therefore, the data were filtered using AlgPred v. 2.0 to predict which allergens in genetically related species might be potential allergens in *Anisakis simplex*. This was performed using bioinformatic analyses based on hybrid scores (ML Score, MERCI Score, BLAST Score) with a threshold value of 1.0 to predict highly allergenic proteins.

ID	ML Score	MERCI Score	BLAST Score	Hybrid Score	Prediction	Protein Names	Gene Names
A0A0M3J693	0.64	0.5	0.5	1.64	Allergen	Tropomyosin	ASIM_LOCUS2926
A0A3P6NAG0	0.46	0.5	0.5	1.46	Allergen	Myosin tail domain-containing protein	ASIM_LOCUS3260
A0A0M3K4R0	0.45	0.5	0.5	1.45	Allergen	Tubulin alpha chain	ASIM_LOCUS15358
A0A0M3KB72	0.29	0.5	0.5	1.29	Allergen	Tubulin_C domain-containing protein	ASIM_LOCUS17621
A0A0M3K1E8	0.29	0.5	0.5	1.29	Allergen	arginine kinase (EC 2.7.3.3)	ASIM_LOCUS14097
A0A3P6R6D8	0.16	0.5	0.5	1.16	Allergen	Tubulin alpha chain	ASIM_LOCUS16522
A0A0M3KE55	0.62	0	0.5	1.12	Allergen	CX9C domain-containing protein	ASIM_LOCUS18653
A0A3P6N442	0.6	0.5	0	1.1	Allergen	Myosin tail domain-containing protein	ASIM_LOCUS2417
A0A158PPL0	0.08	0.5	0.5	1.08	Allergen	Tubulin alpha chain	ASIM_LOCUS14373
A0A0M3IXZ4	0.07	0.5	0.5	1.07	Allergen	Apolipophorins	ASIM_LOCUS19
A0A3P6NTK3	0.56	0	0.5	1.06	Allergen	protein disulfide-isomerase (EC 5.3.4.1)	ASIM_LOCUS4939
A0A3P6P7Q2	0.56	0	0.5	1.06	Allergen	Myosin tail domain-containing protein	ASIM_LOCUS6635

**Table 5:** Subcellular localization of allergens in *Anisakis simplex*. Using the DeepLoc v. 2.1 bioinformatics server, the localization of these putative allergens was predicted, showing that 10 of them are cytoplasmic and 2 are extracellular (A0A0M3KE55 and A0A0M3IXZ4).

ID	Protein Names	Localizations	Signals	Membrane Types
A0A0M3J693	Tropomyosin	Cytoplasm		Soluble
A0A3P6NAG0	Myosin tail domain-containing protein	Cytoplasm	Nuclear localization signal	Soluble
A0A0M3K4R0	Tubulin alpha chain	Cytoplasm	Nuclear localization signal	Soluble
A0A0M3KB72	Tubulin_C domain-containing protein	Cytoplasm	Nuclear export signal	Soluble
A0A0M3K1E8	arginine kinase (EC 2.7.3.3)	Cytoplasm		Soluble
A0A3P6R6D8	Tubulin alpha chain	Cytoplasm	Nuclear export signal	Soluble
A0A0M3KE55	CX9C domain-containing protein	Extracellular|Endoplasmic reticulum	Signal peptide	Soluble
A0A3P6N442	Myosin tail domain-containing protein	Cytoplasm	Nuclear localization signal	Soluble
A0A158PPL0	Tubulin alpha chain	Cytoplasm	Peroxisomal targeting signal	Soluble
A0A0M3IXZ4	Apolipophorins	Extracellular	Signal peptide	Soluble
A0A3P6NTK3	protein disulfide-isomerase (EC 5.3.4.1)	Cytoplasm		Soluble
A0A3P6P7Q2	Myosin tail domain-containing protein	Cytoplasm	Nuclear localization signal	Soluble

**Table 6:** In silico analysis of peptide binding to MHC-II ligands. Allergenic proteins from *Anisakis simplex* were mapped to human MHC-II ligands using the NetMHCIIpan-4.3 server. The analysis considered a percentile rank of 0.02, peptide lengths of 15 mer, and relevant human MHC-II alleles from the DR, DQ, and DP gene loci. The protein name and ID are from Uniprot. The pTM (predicted template matching score) and ipTM (predicted interface template matching score) are from AlphaFold analysis.

*A. simplex* Allergen (Uniprot ID)	Protein Name (Uniprot)	*A. simplex* Allergen Peptides (NetMHCIIpan 4.3)	MHC II Alelle (NetMHCIIpan 4.3)	% Rank (NetMHCIIpan)	ipTM (Alphafold)	pTM (Alphafold)
A0A3P6N442	Myosin tail domain-containing protein	NLHNEIAEWEDKYDS	HLA-DQA10102-DQB10502	0.013598	0.55	0.64
A0A3P6N442	Myosin tail domain-containing protein	LHNEIAEWEDKYDSV	HLA-DQA10102-DQB10502	0.009849	0.54	0.63
A0A3P6N442	Myosin tail domain-containing protein	HNEIAEWEDKYDSVN	HLA-DQA10101-DQB10501	0.018159	0.58	0.66
			HLA-DQA10102-DQB10502	0.008021		
			HLA-DQA10105-DQB10501	0.018159		
A0A3P6N442	Myosin tail domain-containing protein	NEIAEWEDKYDSVNK	HLA-DQA10102-DQB10502	0.018927	0.55	0.63
A0A158PPL0	Tubulin alpha chain	KYVPRAIYVDLEPTV	HLA-DQA10501-DQB10201	0.019068	0.47	0.58
A0A3P6R6D8	Tubulin alpha chain	LEHGIQPDGKLPSDK	DRB1_0301	0.008703	0.72	0.74
A0A3P6R6D8	Tubulin alpha chain	EHGIQPDGKLPSDKQ	DRB1_0301	0.005913	0.69	0.72
A0A0M3IXZ4	Apolipophorins	DSSVIFSPKGYIPRG	DRB1_1401	0.013456	0.68	0.72
A0A0M3IXZ4	Apolipophorins	KDGKKFLAKINLPDD	DRB1_0801	0.006986	0.71	0.73
A0A0M3IXZ4	Apolipophorins	DGKKFLAKINLPDDD	DRB1_0801	0.004091	0.66	0.71
A0A0M3IXZ4	Apolipophorins	GKKFLAKINLPDDDA	DRB1_0801	0.003686	0.65	0.7
A0A0M3IXZ4	Apolipophorins	KKFLAKINLPDDDAK	DRB1_0801	0.009572	0.66	0.7
A0A0M3IXZ4	Apolipophorins	LAEVSVEKVDPQLKS	DRB4_0101	0.017142	0.76	0.78
A0A0M3IXZ4	Apolipophorins	MKNAIEVDYRGKKST	DRB1_0301	0.011058	0.73	0.76
A0A0M3IXZ4	Apolipophorins	KNAIEVDYRGKKSTV	DRB1_0301	0.00854	0.72	0.75
A0A0M3IXZ4	Apolipophorins	PQKIDLDVKSQKFDP	DRB1_0301	0.010519	0.7	0.73
A0A0M3K4R0	Tubulin alpha chain	PKDVNSAIATIKTKR	HLA-DPA10201-DPB11401	0.01517	0.75	0.77
A0A0M3KB72	Tubulin_C domain-containing protein	PKDVNAAIATIKTKR	HLA-DPA10201-DPB10301	0.009263	0.76	0.77
			HLA-DPA10201-DPB11401	0.004646		
			HLA-DPA10202-DPB11901	0.019908		
A0A0M3KB72	Tubulin_C domain-containing protein	KDVNAAIATIKTKRS	HLA-DPA10201-DPB11401	0.007915	0.79	0.8
A0A158PPL0	Tubulin alpha chain	DVNAAIATIKTKRGI	HLA-DPA10201-DPB11401	0.019983	0.77	0.78
A0A0M3IXZ4	Apolipophorins	VKRIIDALLQKIPRG	HLA-DPA10202-DPB10202	0.011309	0.82	0.81
A0A0M3IXZ4	Apolipophorins	GRTKYTLTAKTLAGS	HLA-DPA10201-DPB10901	0.015979	0.79	0.8
A0A0M3IXZ4	Apolipophorins	CTEINFIVKNFQSNI	HLA-DPA10103-DPB10201	0.014465	0.79	0.79
A0A0M3IXZ4	Apolipophorins	TEINFIVKNFQSNIG	HLA-DPA10103-DPB10201	0.00436	0.78	0.78
A0A0M3IXZ4	Apolipophorins	EINFIVKNFQSNIGC	HLA-DPA10103-DPB10201	0.007969	0.79	0.79
A0A0M3IXZ4	Apolipophorins	KEPFELMLKNTKSGN	HLA-DPA10202-DPB11901	0.006357	0.78	0.78
A0A0M3IXZ4	Apolipophorins	EPFELMLKNTKSGNR	HLA-DPA10202-DPB11901	0.012887	0.75	0.77

**Table 7:** Primers used to identify different members of the Anisakidae family.

Species	Primers Sequences
*Anisakis physeteris*	APY (5′-GGCTGGTTGATGAACTGTTG-3′)
*Pseudoterranova*	PD (5′-CGAGTACTTTTTATGGTCGTGAAGT-3′)
*Anisakis simplex sensu stricto*	AC (5′-GACATTGTTATTTCATTGTATGTGTTGAAAATG-3′)
*Contracaecum osculatum*	COS (5′-TGATATGCTTGAAAGGCAGG-3′)
*Hysterothylacium aduncum*	HAD (5′-GCCTTCCATATGCGCGTATA-3′)
*Anisakis pegreffii*	APE1 (5′-GAGCAGCAGCTTAAGGCAGAGGC-3′) & APE2 (5′-GAGCAGCAGCTTAAGGCAGATGC-3′).

## Data Availability

The original contributions presented in this study are included in the article/Supplementary Materials. Further inquiries can be directed to the corresponding authors.

## Supplementary Materials

The following supporting information can be downloaded at: https://www.mdpi.com/article/10.3390/ijms27135922/s1.
